# Indicators of improved gestation housing of sows. Part II: Effects on physiological measures, reproductive performance and health of the offspring

**DOI:** 10.1017/awf.2023.48

**Published:** 2023-07-26

**Authors:** Martyna E Lagoda, Keelin O’Driscoll, Maria C Galli, José J Cerón, Alba Ortín-Bustillo, Joanna Marchewka, Laura A Boyle

**Affiliations:** 1 Pig Development Department, Animal & Grassland Research & Innovation Centre, Teagasc Moorepark, Fermoy, Co Cork, Ireland; 2 Institute of Genetics and Animal Biotechnology of the Polish Academy of Sciences, Department of Animal Behaviour, ul. Postępu 36A, Jastrzębiec 05-552; 3 Department of Animal Medicine, Production and Health, University of Padova, Viale dell’Università 16, 35020, Legnaro (PD), Italy; 4 Interdisciplinary Laboratory of Clinical Analysis of the University of Murcia (Interlab-UMU), Regional Campus of International Excellence ‘Campus Mare Nostrum’, University of Murcia, Campus de Espinardo s/n, 30100 Murcia, Spain

**Keywords:** animal welfare, chronic stress, enrichment, piglet, pregnancy, prenatal

## Abstract

Prenatal stress is the mechanism through which poor welfare of pregnant sows has detrimental effects on the health and resilience of their piglets. We compared two gestation housing systems (IMPROVED versus [conventional] CONTROL) in terms of sow stress and welfare indicators and sought to determine whether potential benefits to the sows would translate into improved offspring health. Sows were mixed into 12 stable groups (six groups per treatment, 20 sows per group) 29 days post-service in pens with free-access, full-length individual feeding/lying-stalls. CONTROL pens had fully slatted concrete floors, with two blocks of wood and two chains suspended in the group area. IMPROVED pens were the same but with rubber mats and manila rope in each stall, and straw provided in three racks in the group area. Saliva was collected from each sow on day 80 of pregnancy and analysed for haptoglobin. Hair cortisol was measured in late gestation. Sows’ right and left eyes were scored for tear staining in mid lactation and at weaning. Numbers of piglets born alive, dead, mummified, and total born were recorded. Piglets were weighed and scored for vitality and intra-uterine growth restriction (IUGR) at birth. Presence of diarrhoea in farrowing pens was scored every second day throughout the suckling period. IMPROVED sows had lower haptoglobin levels and tear-stain scores during lactation. IMPROVED sows produced fewer mummified piglets, and these had significantly lower IUGR scores, and scored lower for diarrhoea than piglets of CONTROL sows. Hence, improving sow welfare during gestation improved the health and performance of their offspring.

## Introduction

Sub-optimal housing and management pose risks for chronic stress in sows which negatively affects their welfare, health and productivity (Merlot *et al.* 2013; Martinez-Miro *et al.* 2016; Lagoda *et al.* 2022). Moreover, the detrimental effects of chronic stress can extend beyond the sow to compromise the welfare and resilience of her offspring through the process of prenatal stress (Braastad 1998; Parada Sarmiento *et al.* 2021). Prenatally stressed piglets (for example, piglets born to sows subjected to social stress resulting from mixing with unfamiliar individuals; Rault *et al.* 2013) show impaired stress-coping ability and altered behaviour (i.e. longer latency to the first escape attempt) in contrast to non-prenatally stressed piglets (Weinstock 1997; Rault *et al.* 2013). They also have reduced immunity, and are consequently more susceptible to disease (Tuchscherer *et al.* 2002; Albernaz-Gonçalves *et al.* 2022), particularly diarrhoea, both during the suckling and post-weaning periods (Friendship 2020). Diarrhoea is the main reason for antibiotic use in young pigs (Albernaz-Gonçalves *et al.* 2021). As antimicrobial treatment for diarrhoea is applied to all piglets in the pen, it is a major contributor to antibiotic use in pig production (Campbell *et al.* 2013; Xu *et al.* 2018; Albernaz-Gonçalves *et al.* 2021). This, combined with the on-farm prophylactic use of in-feed antibiotics, is a risk factor for the development of antimicrobial resistance (Koju *et al.* 2022; O’Neill 2022).

The threat of antimicrobial resistance generated interest in the development of ‘drug-free’ methods of improving immunity to optimise animal health (Xu *et al.* 2018; Dawkins 2019; Albernaz-Gonçalves *et al.* 2022). Dawkins (2019) suggests the potential of good management and higher standards of welfare to act as preventative medicine. In line with this, van Dixhoorn *et al*. (2016) showed that pigs housed in enriched pens (social and environmental enrichment) cleared viral PRRSV RNA from blood serum faster, developed fewer lung lesions, and had lower levels of pneumonia compared to pigs from barren pens. In addition, pigs in enriched pens showed less stress-related behaviour and differed immunologically and clinically from pigs in barren pens. Moreover, sows housed in larger pens with deep straw bedding maintained to a good standard of hygiene had lower granulocyte counts (Merlot *et al*. 2019), and lower concentrations of blood haptoglobin (Merlot *et al.* 2017), indicative of lower levels of microbial infection, inflammation and stress. These studies demonstrate the association between improved welfare and health (Fraser 2009).

Furthermore, maternal and prenatal stress are linked, so improving sow welfare during gestation could reduce prenatal stress levels acting on developing offspring *in utero* (Tuchscherer *et al.* 2002; Kranendonk *et al.* 2008), translating into improved health and resilience of piglets early (Rault *et al.* 2013), and potentially later (Jarvis *et al.* 2006; Merlot *et al.* 2017, 2019) in life. For instance, sows on deep straw bedding and in larger pens (Merlot *et al.* 2017; Quesnel *et al.* 2019a), or provided with enrichment (Quesnel *et al.* 2019b) produced offspring with better health. Quesnel *et al.* (2019a) demonstrated compromised tissue maturity (lighter gut and lower glycogen content of the longissimus muscle) in piglets born to sows from conventional, non-enriched pens, compared to piglets born to sows from enriched pens. In addition, Quesnel *et al.* (2019a) and Merlot *et al.* (2017) recorded lower mortality 12 h after birth, and lower pre-weaning mortality for litters of sows housed in enriched pens during pregnancy (compared to barren pens). However, these findings should be interpreted with caution, as in some instances prenatal stress can be advantageous by preparing offspring for their future environment. For instance, prenatal stress resulting from inadequate maternal nutrition can improve offspring resilience by modifying their metabolic phenotype to better use available resources later in life, and thus ensure greater resilience and survivability (Gonzalez-Bulnes *et al.* 2016).

Although additional space or deep straw bedding certainly improves sow welfare (Merlot *et al*. 2017; Quesnel *et al.* 2019a,b), such approaches are potentially disruptive to the management or operation of the farm (Winkel *et al.* 2020; Lagoda *et al.* 2023). In our sister paper (Lagoda *et al.* 2023), we demonstrated that the implementation of a number of smaller, incremental improvements to conventional gestation housing (rubber mats, manila ropes and foraging substrates provided in rooting towers) can improve sow welfare. In addressing psychological and physical stressors experienced by pregnant sows, levels of oral stereotypies and tear-stain scores were lower, which is likely indicative of reduced chronic stress.

Chronic stress compromises immune function, and potentially contributes to a chronic inflammatory response (Salak-Johnson & McGlone 2007; Xiong *et al.* 2022). Levels of acute phase proteins, such as haptoglobin, are increased during systemic inflammation, and hence can be used as stress markers (Wang *et al.* 2001), in addition to traditional measures of cortisol. Specifically, haptoglobin in pigs is a ‘moderate slow’ acute phase protein which can be used to assess the immune status, and thus chronic stress under different conditions, over a prolonged period (Millet *et al.* 2005; Murata 2007; Cerón *et al.* 2022). For example, pigs housed in an organic production system showed lower haptoglobin levels at slaughter, indicating more stress resistance compared to pigs from a conventional production system (Millet *et al.* 2005). Thus, haptoglobin can reflect levels of chronic stress (Millet *et al.* 2005).

The objective of this study was to determine if improvements to welfare associated with less chronic stress during gestation would translate into a reduced inflammatory response in sows in late pregnancy, and whether this, in turn, could improve reproductive performance and contribute to better health of the offspring.

## Materials and methods

### Ethical approval

Experimental work was authorised by the Teagasc Animal Ethics Committee (Approval no TAEC 2020-266, and TAEC 2020-267). No invasive measures were employed in this farm-based trial. Experimental animals were monitored twice daily by farm staff and researchers, and any sick or injured sows or piglets were treated immediately or, if necessary, removed from the trial.

### Assignment of animals to trial, housing and management

Details regarding the assignment of animals to trial, housing and management, as well as results for the effects of improved gestation housing on chronic stress and welfare indicators are described in Lagoda *et al.* (2023). In summary, the study took place on a 2,000-sow commercial farrow-to-finish farm in County Cork, Ireland, between July 2021 and April 2022. Sows went on trial over two three-week periods (period 1: 2–16 August 2021; period 2: 15–29 November 2021), whereby 40 served sows were enrolled in the study every week, with 20 sows assigned to conventional (CONTROL) and 20 to treatment (IMPROVED) pens. In total, the study used 240 sows of parity 1 to 5 (mean [± SD]; 2.4 [± 1.03]) in six replicates.

**Figure 1.**
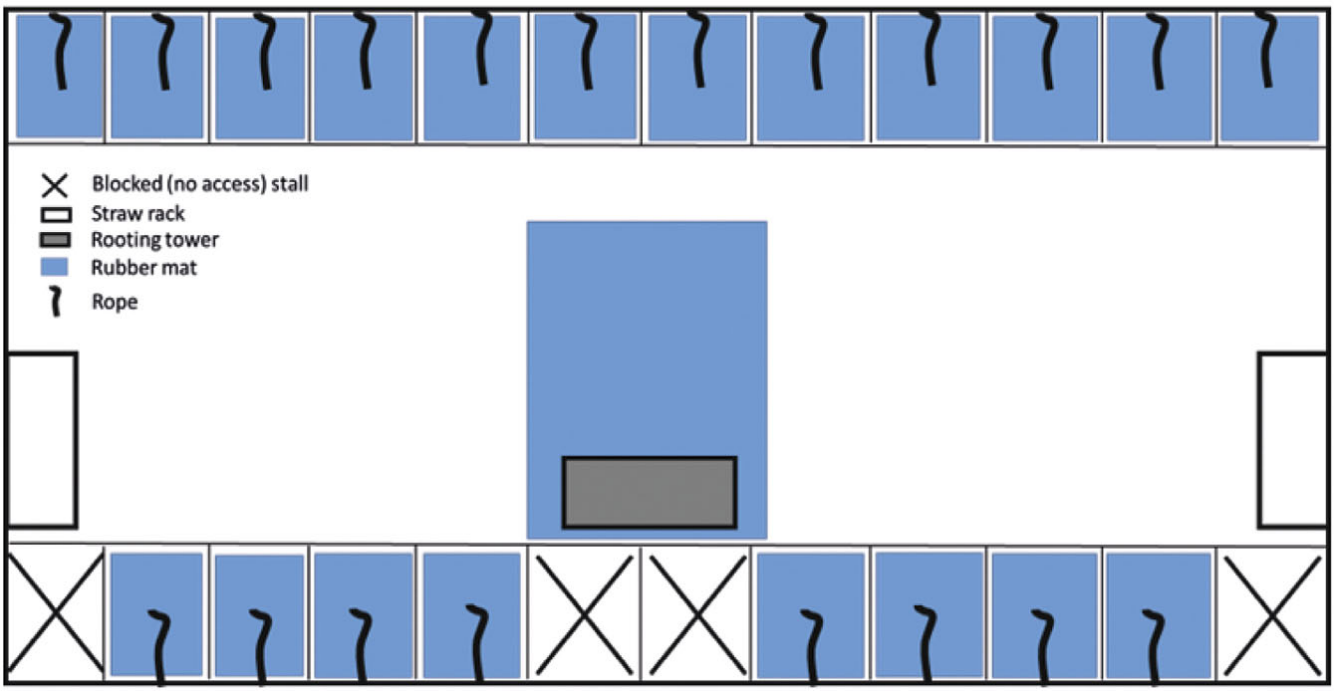
Diagram of the layout and set-up of the IMPROVED pen for pregnant sows.

The experiment started on the day sows were mixed into the gestation pens (day 28.9 [± 0.37] post-insemination). Thereafter, the composition of each group was stable. Each pen had 20 individual, free-access feeding/lying stalls (2.30 × 0.55 m; length ×width), and sows were free to move around the remainder of the pen (7.3 × 7.2 m; roaming area between two rows of feeding stalls: 7.3 × 2.7 m). CONTROL pens had fully slatted concrete floors, two blocks of wood and two chains suspended in the loose area. In replicates four to six, pens also had a rubber toy (Astro 200, EasyFix Rubber Products, Ballinasloe, County Galway, Ireland) suspended from a chain. IMPROVED pens were the same, but with the addition of a length of natural fibre rope (1-m manila rope; Marine Suppliers & Co Ltd, Howth, Co Dublin; replenished every two weeks throughout gestation) suspended from the feed trough within each feeding stall, and straw provided (replenished daily) in three custom-made structures (two straw racks at each end of the pen, and a rooting tower in the middle of the roaming area; Figure 1). Additionally, in the IMPROVED treatment, the slats in each feeding stall, as well as in front of the rooting tower, were covered with rubber mats (EasyFix Rubber Products, Ballinasloe, Co Galway, Ireland).

Sows were fed a liquid diet twice per day and had *ad libitum* access to water via three nipple drinkers at one end of the pen. Sows were transferred into conventional farrowing crates (2.2 × 0.6 m) with fully slatted cast-iron floors and a heated pad for piglets one week prior to farrowing. Piglets were cross-fostered within treatment only, and within the first 24 h of life, after piglet measures (birth weight, intra-uterine growth restriction, IUGR, and vitality) were recorded. Piglets were weaned at approximately 28 days post-farrowing (26 [± 2.3] days).

All measures were performed by a single observer who practiced prior to the beginning of the study until 90% intra-observer repeatability was achieved. The observer was blind to treatments for the measures carried out in the farrowing rooms.

### Sow measures

#### Salivary haptoglobin

A single saliva sample was collected from each sow on day 79.7 (± 1.21) of pregnancy, between 0800 and 0900h (Kováč *et al.* 2008) by allowing her to chew on a 5 × 2 × 2 cm (length × width × breadth) polypropylene sponge (Esponja Marina, La Griega E Koronis, Madrid, Spain) clipped to a metal rod for approximately 1 min, until thoroughly moist. Moist sponges were placed inside Salivette tubes (Salimetrics) and centrifuged for 5 min at 3,000 rpm. Saliva samples were frozen at –20°C pending analysis in a professional biomarker laboratory (Interdisciplinary Laboratory of Clinical Analysis of the University of Murcia, Spain). Haptoglobin was measured using an in-house assay based on alphaLISA technology and a method described by Ortín-Bustillo *et al.* (2022), with intra- and inter-assay coefficient of variation (CV) < 15%.

#### Hair cortisol

Sows were restrained in the free-access stalls on day 25 of gestation, and then again in late pregnancy (day 109) to collect hair samples. An electric razor was used to shave hair from the dorso-lumbar region, identified by measuring 6.5 cm left and right from the mid-point at the spine marked by the position of the last rib. Hair samples were placed into plastic zip-lock bags, and frozen at −20°C until hair cortisol analysis. Samples of re-grown hair collected in late pregnancy (day 109) were analysed for cortisol concentration.

Hair sample preparation and cortisol extraction were based on the procedure described by Davenport *et al.* (2006), with certain modifications described by Lagoda *et al.* (2021). In brief, hair samples were defrosted for 1 h prior to preparation procedures, then washed by placing 300 mg of hair into a 10 mL polypropylene tube along with 5 mL of isopropanol, and mixing gently on a shaker for 3 min. This was repeated using fresh isopropanol for the second wash. Washed hair samples were left inside the wash tubes and placed inside a protected fume hood to dry overnight. Samples prepared in this way were then individually ground into a fine powder using a Retsch mixing mill (MM200; 10 mL stainless steel grinding jars, single 12-mm stainless steel grinding ball) for 4 min at 25 Hz. Approximately 50 mg of ground hair was weighed and placed in a 2 mL tube along with 1 mL of methanol, which was incubated for 24 h at room temperature with constant gentle agitation (approximately 95 rpm) for cortisol extraction. Following the 24-h incubation period, we removed 0.6 mL of the cortisol extract in methanol (taking care not to disturb the settled hair powder at the bottom of the tube) using an Eppendorf pipette and transferred to a clean 1.5 mL tube for methanol evaporation, which was performed using a stream of nitrogen gas at 38°C. Cortisol extracts were frozen at −20°C pending EIA analysis. Extracted samples of cortisol were analysed using Salimetrics® Expanded Range, High Sensitivity Salivary Cortisol EIA kit. Frozen extract samples along with the EIA kit were brought to room temperature 1.5 h prior to being reconstituted with 0.4 mL of phosphate buffer (assay diluent) provided with the EIA kit. Reconstituted extracts (n = 159) were analysed for cortisol concentration levels in duplicate using five assays, following the protocol provided with the EIA kit. Inter- and intra-assay CV were 11.5 and 15.9%, respectively.

#### Tear staining (Chromodacryorrhea)

We scored the area of tear staining for the left and right eye when sows were in mid lactation and at weaning, according to a scale (Table 1) developed by DeBoer *et al.* (2015).

**Table 1.** Description of the scores, developed by DeBoer *et al.* (2015) used in the assessment of tear staining in sows

Description of tear stain	Score
No visible stains	0
Barely detectable stains, not extending below eyelid	1
Visible stain, < 50% of the size of the eye	2
Visible stain, 50–100% of the size of the eye	3
Visible stain, > 100% of the size of the eye, but not extending below the mouth line	4
Visible stain, extending below the mouth line	5

#### Reproductive performance

Sow reproductive performance was recorded by the research team and included the following measures: number of piglets born alive, born dead, mummified, and total born.

### Offspring measures

#### Birth weight, vitality and intra-uterine growth restriction (IUGR)

Piglets were weighed and scored for vitality and IUGR at birth. Vitality was scored according to criteria shown in Table 2, modified from Schmitt *et al.* (2019) and Rooney *et al.* (2020). The summation of scores for each criterion yielded a total vitality score, with the maximum (best) possible score of three per piglet. The degree of IUGR was estimated by visually scoring the presence/absence of nose wrinkles, cone-shaped head, and bulging eyes, based on a method from Hales *et al.* (2013). For all three measures, a piglet scored 0 if the trait was absent, and 1 if it was present; therefore, the maximum (worst) total IUGR score a piglet could receive was 3.

**Table 2.** Vitality scoring system used for piglets at birth (Schmitt et al. 2019; Rooney *et al*. 2020)

Vitality indicators	Vitality score	
	0	1
Reaction to handling - Vocalisation & escape	Piglet does not scream during handling, or piglet does not attempt to escape during handling	Piglet screams, or piglet attempts to escape
Muscle tone	Leg muscles are soft when pressed against handler’s palm	Leg muscles are firm and piglet pushes back against handler’s palm
Initial position on return to the farrowing pen	Piglet is on its back or lies on its side without trying to right itself (for > 20 s)	Piglet is immediately up on its four legs and moves away (within 20 s)

#### Diarrhoea

Presence of diarrhoea in the farrowing pen was scored approximately every second day throughout lactation (always starting on the Monday after farrowing, until the day of weaning; n = 10 scores per litter). This allowed us to distinguish fresh faeces from those present on the previous scoring day, according to criteria shown in Table 3, based on a method from Casey *et al.* (2007) and Marquardt *et al.* (1999) (0 = normal faeces, to 3 = severe diarrhoea). Scores were summed to yield a total diarrhoea score (TDS) per pen per sow per litter throughout lactation.

**Table 3.** Scoring system used for pen diarrhoea level assessment throughout lactation, adapted from Marquardt *et al*. (1999) and Casey *et al*. (2007)

Description	Faecal score
Normal – dry, pelleted faeces	0
Soft – soft faeces, with shape	1
Mild diarrhoea – very soft or viscous liquid faeces	2
Severe diarrhoea – watery faeces, or with blood	3

#### Statistical analysis

SAS v9.4 was used for all statistical analyses (SAS Inst Inc, Cary, NC, USA) with sow as the experimental unit. Differences were reported when *P* ≤ 0.05. Residuals were checked for normality by examination of histograms, quantile-quantile and normal distribution plots using the univariate procedure. Degrees of freedom were estimated using the Kenward-Rogers adjustment, and *P*-values adjusted using the Tukey-Kramer adjustment where mixed models were used. Data are presented as least square (LS) means and standard errors (SE).

All general linear models included the fixed effects of treatment and replicate, and pen as a random effect. The model for piglet birth weight also included the interactive effect of treatment and piglet sex, the repeated effect of piglet to allow for repeated measures, and sow as the subject. Covariance structure was selected on the basis of best fit, using the minimum finite-sample corrected Akaike Information Criteria (AIC).

Salivary haptoglobin, hair cortisol, piglet birth weight, total diarrhoea score, the number of piglets born alive, and the total number born were analysed using general linear models (PROC MIXED), while the number of piglets born dead and mummified were analysed using PROC GLIMMIX (with Poisson set as the distribution, and no offset command).

The Mann-Whitney test (PROC Npar1Way) was used to compare tear stains for both the right and left eyes of sows from CONTROL and IMPROVED pens, in mid lactation and at weaning. Right and left eyes were analysed separately, as previous work showed differences in tear staining for both eyes in response to stressors (DeBoer *et al.* 2015). *P*-values were adjusted *post hoc* using the Bonferroni adjustment to account for multiple comparisons. The Mann-Whitney test (PROC Npar1Way) was also used to compare vitality scores, while a Chi-squared test was used to compare IUGR scores of piglets born to sows from CONTROL and IMPROVED pens.

## Results

### Effect of treatment on sow measures

There were higher concentrations of haptoglobin in the saliva of CONTROL compared to IMPROVED sows (*P* = 0.007; Table 4). There was no effect of treatment on concentrations of cortisol in the hair (*P* = 0.438; Table 4).

**Table 4.** Differences (least square means (± SE]) in saliva haptoglobin, hair cortisol, and reproductive performance of 240 sows housed in either conventional (CONTROL; n = 120) or IMPROVED (n = 120) pens, as well as differences in the measures recorded for their offspring (birth weight, diarrhoea score)

Variable	CONTROL	IMPROVED	*P*-value
*Sow measures*			
Haptoglobin (ng ml^–1^)	671.77 (± 53.91)	463.36 (± 54.14)	0.007
Hair cortisol (μg dL^–1^)	0.09 (± 0.01)	0.10 (± 0.01)	0.438
Born alive	14.55 (± 0.28)	14.88 (± 0.29)	0.417
Born dead	1.43 (± 0.11)	1.19 (± 0.10)	0.113
Mummified	0.40 (± 0.06)	0.23 (± 0.04)	0.013
Total born	16.45 (± 0.30)	16.34 (± 0.31)	0.800
*Piglet measures*			
Birth weight (kg)	1.40 (± 0.02)	1.45 (± 0.02)	0.069
Total diarrhoea score (TDS)	5.43 (± 0.23)	3.72 (± 0.23)	< 0.001

**Figure 2.**
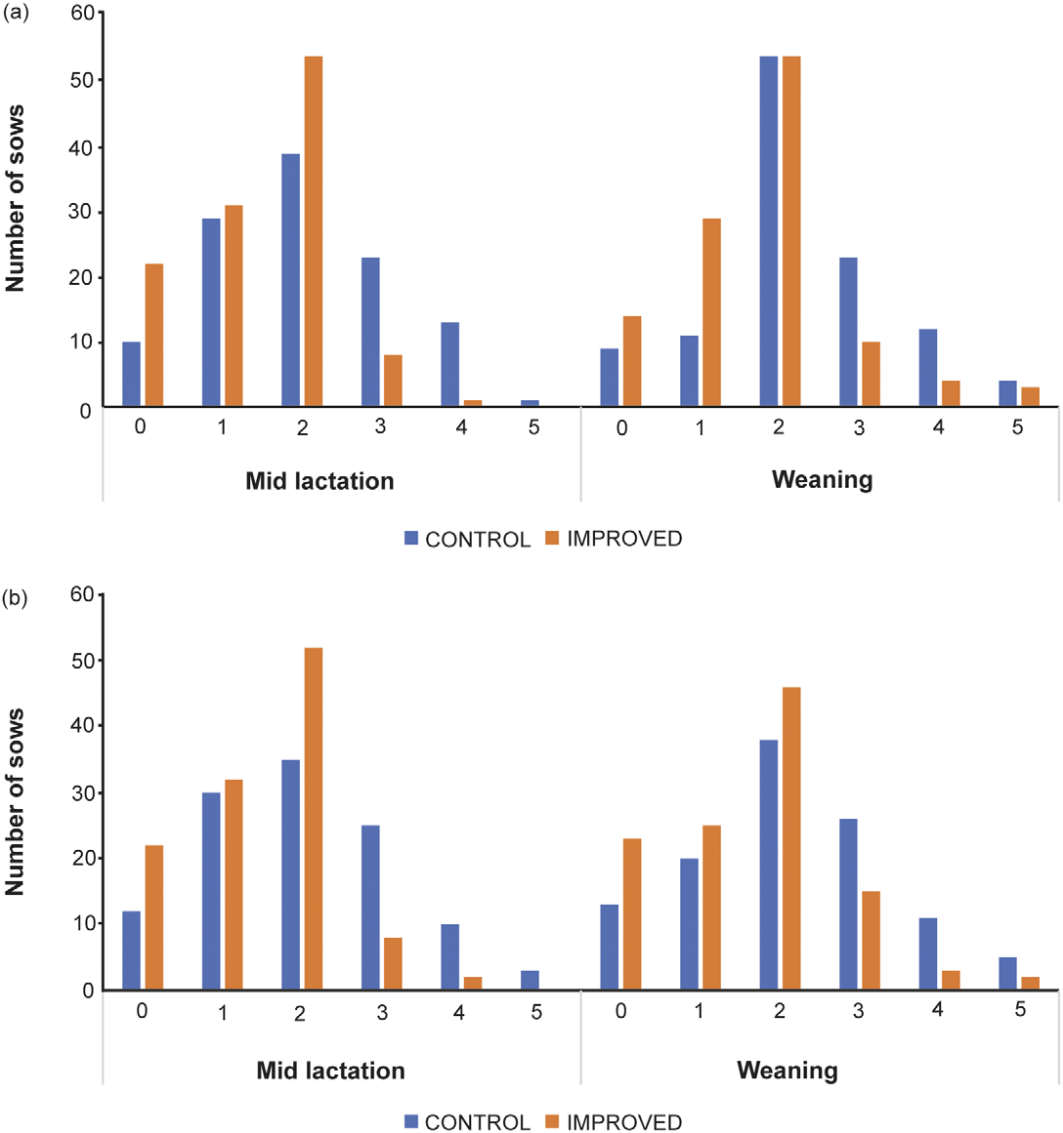
The number of sows per tear-stain score category around the (a) right and (b) left eyes in mid lactation and at weaning, for the two treatment groups.

Sows in the IMPROVED pens had lower (mean [± SD]) tear-stain scores in mid lactation (Right eye: 1.4 [± 0.91]; Left eye: 1.4 [± 0.94]) and at weaning (Right eye: 1.7 [± 1.07]; Left eye: 1.6 [± 1.13]) compared to CONTROL sows (Mid lactation, right eye: 2.0 [± 1.16], left eye: 2.0 [± 1.22]; Weaning, right eye: 2.3 [± 1.14], left eye: 2.2 [± 1.28]; All *P* ≤ 0.005; Figure 2).

While there was no effect of treatment on the number of piglets born alive, born dead and the total number born, CONTROL sows gave birth to more mummified piglets compared to IMPROVED sows (*P* = 0.013; Table 4).

## Effect of treatment on offspring measures

Piglets born to CONTROL sows tended to have lower weights at birth compared to those born to IMPROVED sows (*P* = 0.069; Table 4). There was no effect of treatment on piglet vitality at birth (*P* > 0.05), however there was an effect of treatment on IUGR scores. Piglets born to CONTROL sows had higher IUGR scores compared to those born to IMPROVED sows (*P* = 0.007; Table 5).

**Table 5.** Number of piglets per intra-uterine growth restriction (IUGR) score category at birth

IUGR score	CONTROL	IMPROVED
0	1,557	1,555
1	56	31
2	4	2
3	0	0

Finally, there was an effect of treatment on farrowing pen diarrhoea scores during the suckling period. Pens of piglets born to CONTROL sows had higher total diarrhoea scores compared to pens of piglets born to IMPROVED sows (*P* < 0.001; Table 4). Table 6 shows the number of farrowing pens that received a score of ≥ 2 in each treatment, along with the range in scores per day of lactation on which the pens were scored. Only one farrowing pen was scored 3 throughout the entire study.

**Table 6.** Number of farrowing pens scored ≥ 2 in each treatment with the range in scores per day of lactation on which they were scored

	CONTROL		IMPROVED	
Day of lactation	Pen (n) with score ≥ 2	Diarrhoea score range	Pen (n) with score ≥ 2	Diarrhoea score range
5	53	0 to 3	25	0 to 2
7	39	0 to 2	28	0 to 2
9	11	0 to 2	10	0 to 2
12	8	0 to 2	6	0 to 2
14	10	0 to 2	5	0 to 2
16	10	0 to 2	4	0 to 2
19	6	0 to 2	2	0 to 2
21	4	0 to 2	7	0 to 2
23	13	0 to 2	4	0 to 2
26	9	0 to 2	6	0 to 2

## Discussion

There is growing evidence that chronic stress experienced by pregnant sows in conventional housing systems contributes to prenatal stress, which can be detrimental to their offspring (Braastad 1998; Albernaz-Gonçalves *et al.* 2022). Improving sow welfare is therefore an important step towards ensuring good health and resilience of piglets (Kapoor *et al.* 2006). Our findings support this hypothesis with improved sow welfare during pregnancy (described in Lagoda *et al.* 2023) translating to piglets with a tendency towards higher birth weights, significantly lower IUGR scores, and less diarrhoea during the suckling period.

As a likely consequence of the lower levels of chronic stress during gestation (Wang *et al.* 2001), sows in the improved pens had lower concentrations of salivary haptoglobin in late pregnancy. Saliva samples were taken on day 80 of pregnancy, encompassing seven weeks of exposure to the different housing systems for the sows in each treatment. This was obviously a sufficient amount of time for haptoglobin levels to diverge between the two treatments. This divergence indicates lower levels of systemic inflammation and disease in sows in the improved pens (Wang *et al.* 2001), and suggests improved health and better stress-coping abilities (Millet *et al.* 2005; Murata 2007; Cerón *et al.* 2022). This is in line with previous work, whereby finisher pigs housed in an organic system had lower haptoglobin levels compared to pigs housed in a conventional system (Millet *et al.* 2005). Similarly, growing pigs kept in enriched housing had lower levels of haptoglobin and were less affected by stress induced by a regrouping test compared to pigs housed in barren pens (Reimert *et al.* 2014).

It is possible that the consumption of straw also had a positive impact on the digestive/gut health of sows in the improved pens, protecting against inflammation, and contributing to lower haptoglobin levels. Indeed, Kobek-Kjeldager *et al.* (2022) hypothesised that certain dietary fibres stimulate beneficial gut microbiota that protect against inflammation and improve stress resilience. Although we cannot discern the extent of the contribution made by the different enrichment substrates or the rubber mats to sow welfare, it is likely that straw played a major role. This is based on the relevance of this enrichment type to highly motivated sow behaviours, as well as the benefits it provides for gut health and satiety (Tuyttens 2005; Stewart *et al.* 2008). Moreover, chewing straw or ropes may influence saliva production, and therefore improve dental/buccal health (as described in humans; Haigh *et al.* 2010). This could also have contributed to the reduction seen in haptoglobin levels in the improved sows.

We found no difference in the concentration of hair cortisol between treatments. This is contrary to our findings on haptoglobin, tear staining, and stereotypical behaviour which all point to reduced stress in sows in improved pens (Lagoda *et al.* 2023). It is possible that the methodology we employed was flawed or inaccurate, that hair cortisol does not accurately reflect chronic stress, or that the levels of stress were simply not divergent enough between the two treatments to cause a difference. We employed the shave/re-shave method (first shave on day 30, then re-shave performed in late pregnancy), as it more accurately determines cortisol concentrations, and thus stress corresponding to approximately the last two-thirds of the pregnancy. The collection site can impact on concentrations of cortisol found in hair (Heimbürge *et al.* 2019; Lagoda *et al.* 2021). Therefore, based on previous research, we selected the dorso-lumbar region as the most appropriate site for hair collection to ensure adequate measurement of cortisol concentrations (Casal *et al.* 2017; Otten, *et al.* 2020; Lagoda *et al.* 2021). Nevertheless, cortisol concentrations in hair are also influenced by cleanliness (Davenport *et al.* 2006; Meyer & Novak 2012; Heimburge *et al.* 2019). For instance, endogenous hair cortisol concentrations may be altered by exogenous cortisol entering the hair shaft by diffusion from urine, faeces and saliva, which commonly contaminate the outside of sow hair in on-farm settings (Otten *et al.* 2020). Working within the limits of a commercial farm it was not possible to control for cleanliness of sow hair during gestation.

Previous authors consider hair a suitable medium for quantifying levels of chronic stress in cattle and sheep (Salaberger *et al.* 2016; Heimburge *et al.* 2020) due to the long-term accumulation of cortisol within the shaft (Davenport *et al.* 2006; Meyer & Novak 2012; Heimbürge *et al.* 2019). However, in pigs, the value of hair cortisol is less clear (Lagoda *et al*. 2021). For instance, following a period of ACTH injections in cattle and pigs, there were differences in hair cortisol between treatments for cattle, but not for pigs (Heimburge *et al.* 2020). It is possible that this is due to a lower systemic cortisol response following an ACTH challenge, or a faster rate at which ACTH is metabolised in pigs (Heimburge *et al.* 2020).

Sows kept in the improved housing environment during pregnancy also had somewhat better reproductive performance, with lower numbers of piglets born mummified. However, as this difference was small, its biological significance is questionable. Nonetheless, it further supports our hypothesis of improved health status of those sows (evidenced by lower haptoglobin levels), and is in accordance with Lewis *et al.* (2009) who also found improved reproductive performance in healthier sows. In that study, sows infected with PRRSV gave birth to more mummified piglets (Lewis *et al.* 2009). It is also in line with Hartnett *et al.* (2020) who found fewer piglets born dead to sows reared in female-only groups as gilts. These sows also had lower salivary cortisol concentration, indicating lower stress levels resulting from a more optimal rearing environment, with carry-over benefits for their future performance (Hartnett *et al.* 2020). It is likely that healthy, less-stressed sows redirect fewer energy and metabolic resources away from developmental and maintenance processes such as pregnancy, towards processes aimed at ensuring survival, e.g. to fight off infection (Einarsson *et al.* 2008, Kick *et al.* 2011).

Formation of the skeleton of the pig foetus (calcification) takes place around day 38 to 45 of gestation, and any foetus that dies *in utero* prior to this undergoes reabsorption by the sow (Flowers 2019, 2020). The greater number of mummified piglets born to sows in the control treatment suggests that sows continued to redirect energy and metabolic resources away from the reproductive process in mid gestation. In addition to their reduced health status as reflected in the higher levels of haptoglobin, this could also reflect the higher levels of stress experienced by these sows during gestation (Lagoda *et al.* 2023). It is also in line with the findings of Lagoda *et al.* (2021) who reported a similar negative effect of mid- to late-gestation stress (induced by sustained aggression) in sows on the numbers of mummified piglets.

The improved gestation environment enhanced the welfare of the sows and, in accordance with Dawkins (2019), led to the sows having better health outcomes which extended to their offspring. Piglets born to sows kept in the improved environment during pregnancy tended to be heavier at birth and to have significantly lower IUGR scores compared to piglets born to sows from control pens. This suggests those piglets received more resources from their mother *in utero* (Costa *et al.* 2019; Rooney *et al.* 2020). Perhaps there were more resources available for the reproductive process as a by-product of better health and welfare of the mothers (Kick *et al.* 2011; He *et al.* 2019). Indeed Lagoda *et al.* (2021) found higher IUGR scores in piglets born to sows that suffered higher levels of chronic stress resulting from sustained aggression, while Rooney *et al.* (2020) found the same in piglets born to sows fed low energy diets in late gestation. These findings suggest that maternal stress can have the same impact as protein or energy intake deficits when it comes to foetal development.

Moreover, although there were fewer IUGR piglets born to improved sows, there was no treatment effect on vitality scores. Other research indicates that survivability of IUGR piglets is compromised (Baxter *et al.* 2008) which could be reflected in vitality scores at birth. Indeed, allometric measurements of the piglet body parts in addition to IUGR scores is a more objective method of determining IUGR (Baxter *et al.* 2008) and should be employed in future studies.

Pens of piglets born to sows from the improved environment showed lower diarrhoea scores throughout the suckling period. This likely reflects improved immune function, resilience and health, and provides evidence for the beneficial impact of improved sow welfare and lower chronic stress levels during gestation on offspring outcomes (Albernaz-Gonçalves *et al.* 2022). This is in line with studies by Merlot *et al.* (2016, 2019, 2022) and Quesnel *et al.* (2019a) that showed improved piglet immune function and tissue maturity, and consequently lower mortality during the suckling period when pregnant sows were provided with wood and straw pellets, or a larger pen with deep straw bedding. Specifically in relation to the potential carry-over benefits to offspring of maternal straw consumption, Cheng *et al.* (2018) and Bernardino *et al.* (2016) demonstrated that piglets born to sows fed high fibre diets during pregnancy have altered intestinal microbiota and reduced intestinal permeability, with fewer skin lesions prior to weaning suggesting less agonistic interactions. This is evidence for a longer-term carry-over benefit of improved maternal diet, and hence welfare, on the offspring.

Lactating sows from improved gestation housing had lower tear-stain scores in mid lactation and at weaning, suggesting a beneficial carry-over effect of the gestation housing environment during housing in farrowing crates. This is in agreement with Espejo-Beristain *et al.* (2022) where sows from enriched pens had lower levels of cortisol in hair in late pregnancy, and displayed behaviours indicative of better coping ability during and after farrowing, compared to sows from non-enriched pens. The benefits of the improved gestation housing environment clearly overrode any potential stress arising for the improved sows from the sudden transition from rubber mats to lie on and an enriched environment to barren farrowing crates with cast-iron floors. Straw consumption during pregnancy may have improved digestive health and reduced stress as reflected in the lower tear stains recorded for improved sows during lactation. In turn, less-stressed sows could have contributed to improved piglet health. Kinane *et al.* (2022, 2021) found that sows kept in free-lactation crates had lower tear-stain scores at weaning than those kept in standard crates. Their piglets performed fewer damaging behaviours during suckling and grew faster post-weaning compared to piglets from standard crates, and the authors hypothesised that the reduced maternal stress, even post-farrowing, was a contributing factor.

## Animal welfare implications and conclusion

Overall, these results emphasise the cumulative effectiveness of improvements to the housing environment in addressing both physical and psychological stressors experienced by pregnant sows, with associated benefits for the health and resilience of their offspring. If implemented on-farm, our findings could not only improve sow and piglet welfare but contribute to a reduction in antibiotic use to treat diarrhoea in piglets during the suckling period. This would, in turn, reduce the risk of antimicrobial resistance development and improve economic returns for the farmer resulting from reduced veterinary/medication costs and improved pig performance.

## References

[r1] Albernaz-Gonçalves R, Olmos G and Hötzel MJ 2021 Exploring farmers’ reasons for antibiotic use and misuse in pig farms in brazil. Antibiotics 10: 331. 33809885 10.3390/antibiotics10030331 PMC8004152

[r2] Albernaz-Gonçalves R, Olmos Antillón G and Hötzel MJ 2022 Linking animal welfare and antibiotic use in pig farming—a review. Animals 12: 216. 35049838 10.3390/ani12020216 PMC8773261

[r3] Baxter E, Jarvis S, D’Eath R, Ross D, Robson S, Farish M, Nevison I, Lawrence A and Edwards S 2008 Investigating the behavioural and physiological indicators of neonatal survival in pigs. Theriogenology 69: 773–783. 18242685 10.1016/j.theriogenology.2007.12.007

[r4] Bernardino T, Tatemoto P, Morrone B, Mazza Rodrigues PH and Zanella AJ 2016 Piglets born from sows fed high fibre diets during pregnancy are less aggressive prior to weaning. PLoS ONE 11: e0167363. 10.1371/journal.pone.0167363 PMC5132218 27907173

[r5] Braastad BO 1998 Effects of prenatal stress on behaviour of offspring of laboratory and farmed mammals. Applied Animal Behaviour Science 61: 159–180.

[r6] Campbell JM, Crenshaw JD and Polo J 2013 The biological stress of early weaned piglets. Journal of Animal Science and Biotechnology 4: 1–4. 23631414 10.1186/2049-1891-4-19 PMC3651348

[r7] Casal N, Manteca X, Peña R, Bassols A and Fàbrega E 2017 Analysis of cortisol in hair samples as an indicator of stress in pigs. Journal of Veterinary Behaviour 19: 1–6.

[r8] Casey PG, Gardiner GE, Casey G, Bradshaw B, Lawlor PG, Lynch PB, Leonard FC, Stanton C, Ross RP and Fitzgerald GF 2007 A five-strain probiotic combination reduces pathogen shedding and alleviates disease signs in pigs challenged with *Salmonella enterica* serovar *Typhimurium*. Applied and Environmental Microbiology 73: 1858–1863. 17261517 10.1128/AEM.01840-06 PMC1828830

[r9] Cerón J, Contreras-Aguilar M, Escribano D, Martínez-Miró S, López-Martínez M, Ortín-Bustillo A, Franco-Martínez L, Rubio C, Muñoz-Prieto A and Tvarijonaviciute A 2022 Basics for the potential use of saliva to evaluate stress, inflammation, immune system, and redox homeostasis in pigs. BMC Veterinary Research 18: 1–17. 35227252 10.1186/s12917-022-03176-w PMC8883734

[r10] Cheng C, Wei H, Xu C, Xie X, Jiang S and Peng J 2018 Maternal soluble fiber diet during pregnancy changes the intestinal microbiota, improves growth performance, and reduces intestinal permeability in piglets. Applied and Environmental Microbiology 84: e01047–01018. 29959248 10.1128/AEM.01047-18 PMC6102992

[r11] Costa KA, Marques DBD, de Campos CF, Saraiva A, Guimarães JD and Guimarães SEF 2019 Nutrition influence on sow reproductive performance and conceptuses development and survival: a review about l-arginine supplementation. Livestock Science 228: 97–103.

[r12] Davenport MD, Tiefenbacher S, Lutz CK, Novak MA and Meyer JS 2006 Analysis of endogenous cortisol concentrations in the hair of rhesus macaques. General and Comparative Endocrinology 147: 255–261. 10.1016/j.ygcen.2006.01.005 16483573

[r13] Dawkins M 2019 Animal welfare as preventative medicine. Animal Welfare 28: 137–141. 10.7120/09627286.28.2.137

[r14] DeBoer S, Garner J, McCain R, Lay Jr D, Eicher S and Marchant-Forde J 2015 An initial investigation into the effects of isolation and enrichment on the welfare of laboratory pigs housed in the PigTurn® system, assessed using tear staining, behaviour, physiology and haematology. Animal Welfare 24: 15–27. 10.7120/09627286.24.1.015

[r15] Einarsson S, Brandt Y, Lundeheim N and Madej A 2008 Stress and its influence on reproduction in pigs: a review. Acta Veterinaria Scandinavica 50: 1–8. 19077201 10.1186/1751-0147-50-48 PMC2630310

[r16] Espejo-Beristain G, Ahuja-Aguirre C, Carrasco-García AA, Hernandez-Cruz B and Paredes-Ramos P 2022 Environmental enrichment for primiparous and multiparous pregnant sows and its effect on cortisol and behavior at farrowing and production parameters at weaning. Livestock Science: 105103.

[r17] Flowers W 2019 Sow longevity and neonatal management. *Proceedings of the London Swine Conference* pp 3–9. 26–27 March 2019, London, Ontario, Canada.

[r18] Flowers WL 2020 Reproductive management of swine. Animal Agriculture pp 283–297. Elsevier: London, UK.

[r19] Fraser D 2009 Assessing animal welfare: different philosophies, different scientific approaches. Zoo Biology: Published in affiliation with the American Zoo and Aquarium Association 28: 507–518. 10.1002/zoo.20253 19434682

[r20] Friendship RM 2020 *The Suckling and Weaned Piglet* pp 1227–1230. Wageningen Academic Publishers: Wageningen, The Netherlands.

[r21] Gonzalez-Bulnes A, Astiz S, Ovilo C, Lopez-Bote C, Torres-Rovira L, Barbero A, Ayuso M, Garcia-Contreras C and Vazquez-Gomez M 2016 Developmental origins of health and disease in swine: Implications for animal production and biomedical research. Theriogenology 86: 110–119. 27238437 10.1016/j.theriogenology.2016.03.024

[r22] Haigh BJ, Stewart KW, Whelan JR, Barnett MP, Smolenski GA and Wheeler TT 2010 Alterations in the salivary proteome associated with periodontitis. Journal of Clinical Periodontology 37: 241–247. 20149214 10.1111/j.1600-051X.2009.01525.x

[r23] Hales J, Moustsen V, Nielsen M and Hansen C 2013 Individual physical characteristics of neonatal piglets affect preweaning survival of piglets born in a noncrated system. Journal of Animal Science 91: 4991–5003. 24078619 10.2527/jas.2012-5740

[r24] Hartnett P, Boyle LA and O’Driscoll K 2020 Rearing in female-only groups and dietary mineral supplementation improves sow welfare in the early parities and lifetime performance. Translational Animal Science 4: 176. 10.1093/tas/txaa176 PMC7745001 33367220

[r25] He J, Guo H, Zheng W, Xue Y, Zhao R and Yao W 2019 Heat stress affects fecal microbial and metabolic alterations of primiparous sows during late gestation. Journal of Animal Science and Biotechnology 10: 1–12. 31700622 10.1186/s40104-019-0391-0 PMC6827230

[r26] Heimburge S, Kanitz E and Otten W 2019 The use of hair cortisol for the assessment of stress in animals. General and Comparative Endocrinology 270: 10–17. 10.1016/j.ygcen.2018.09.016 30287191

[r27] Heimburge S, Kanitz E, Tuchscherer A,and Otten W 2020 Is it getting in the hair? Cortisol concentrations in native, regrown and segmented hairs of cattle and pigs after repeated ACTH administrations. General and Comparative Endocrinology 295. 113534. 10.1016/j.ygcen.2020.113534 32540492

[r28] Jarvis S, Moinard C, Robson SK, Baxter E, Ormandy E, Douglas AJ, Seckl JR, Russell JA and Lawrence AB 2006 Programming the offspring of the pig by prenatal social stress: Neuroendocrine activity and behaviour. Hormones and Behavior 49: 68–80. 10.1016/j.yhbeh.2005.05.004 15961089

[r29] Kapoor A, Dunn E, Kostaki A, Andrews MH and Matthews SG 2006 Fetal programming of hypothalamo-pituitary-adrenal function: prenatal stress and glucocorticoids. Journal of Physiology-London 572: 31–44. 10.1113/jphysiol.2006.105254 PMC1779638 16469780

[r30] Kick AR, Tompkins MB and Almond GW 2011 Stress and immunity in the pig. Animal Science Reviews 212: 51–65.

[r31] Kinane O, Butler F and O’Driscoll K 2021 Freedom to grow: improving sow welfare also benefits piglets. Animals 11: 1181. 33924235 10.3390/ani11041181 PMC8074778

[r32] Kinane O, Butler F and O’Driscoll K 2022 Freedom to move: Free lactation pens improve sow welfare. Animals 12: 1762. 35883309 10.3390/ani12141762 PMC9311877

[r33] Kobek-Kjeldager C, Schönherz AA, Canibe N and Pedersen LJ 2022 Diet and microbiota-gut-brain axis in relation to tail biting in pigs: A review. Applied Animal Behaviour Science 246: 105514.

[r34] Koju P, Shrestha R, Shrestha A, Tamrakar S, Rai A, Shrestha P, Madhup SK, Katuwal N, Shrestha A and Shrestha A 2022 Antimicrobial resistance in *E. coli* isolated from chicken cecum samples and factors contributing to antimicrobial resistance in Nepal. Tropical Medicine and Infectious Disease 7: 249. 36136660 10.3390/tropicalmed7090249 PMC9504632

[r35] Kováč G, Tothova C, Nagy O and Seidel H 2008 Acute phase proteins during the reproductive cycle of sows. Acta Veterinaria 58: 459–466.

[r36] Kranendonk G, Mulder EJH, Parvizi N and Taverne MAM 2008 Prenatal stress in pigs: Experimental approaches and field observations. Experimental and Clinical Endocrinology & Diabetes 116: 413–422. 10.1055/s-2008-1065335 18484065

[r37] Lagoda M, O’Driscoll K, Marchewka J, Foister S, Turner S and Boyle L 2021 Associations between skin lesion counts, hair cortisol concentrations and reproductive performance in group housed sows. Livestock Science 246: 104463.

[r38] Lagoda ME, O’Driscoll K, Galli MC, Marchewka J and Boyle LA 2023 Indicators of improved gestation housing of sows. Part I: Effects on behaviour, skin lesions, locomotion and tear staining. Animal Welfare 32. 10.1017/awf.2023.47 PMC10938266 38487409

[r39] Lagoda ME, Marchewka J, O’Driscoll K and Boyle LA 2022 Risk factors for chronic stress in sows housed in groups, and associated risks of prenatal stress in their offspring. Frontiers in Veterinary Science 9. 10.3389/fvets.2022.883154 PMC9039259 35498729

[r40] Lewis C, Torremorell M, Galina-Pantoja L and Bishop S 2009 Genetic parameters for performance traits in commercial sows estimated before and after an outbreak of porcine reproductive and respiratory syndrome. Journal of Animal Science 87: 876–884. 18952741 10.2527/jas.2008-0892

[r41] Marquardt RR, Jin L, Kim J-W, Fang L, Frohlich AA and Baidoo SK 1999 Passive protective effect of egg-yolk antibodies against enterotoxigenic *Escherichia coli* K88+ infection in neonatal and early-weaned piglets. FEMS Immunology & Medical Microbiology 23: 283–288. 10225287 10.1111/j.1574-695X.1999.tb01249.x

[r42] Martinez-Miro S, Tecles F, Ramon M, Escribano D, Hernandez F, Madrid J, Orengo J, Martinez-Subiela S, Manteca X and Ceron JJ 2016 Causes, consequences and biomarkers of stress in swine: an update. BMC Veterinary Research 12: 1–9. 27543093 10.1186/s12917-016-0791-8 PMC4992232

[r43] Merlot E, Calvar C and Prunier A 2016 Influence of the housing environment during sow gestation on maternal health, and offspring immunity and survival. Animal Production Science 57: 1751–1758.

[r44] Merlot E, Calvar C and Prunier A 2017 Influence of the housing environment during sow gestation on maternal health, and offspring immunity and survival. Animal Production Science 57: 1751–1758. 10.1071/An15480

[r45] Merlot E, Meunier-Salaün M-C, Peuteman B, Père M-C, Louveau I, Perruchot M-H, Prunier A, Gardan-Salmon D, Gondret F and Quesnel H 2022 Improving maternal welfare during gestation has positive outcomes on neonatal survival and modulates offspring immune response in pigs. Physiology & Behavior 249: 113751. 10.1016/j.physbeh.2022.113751 35217067

[r46] Merlot E, Pastorelli H, Prunier A, Pere MC, Louveau I, Lefaucheur L, Perruchot MH, Meunier-Salaun MC, Gardan-Salmon D, Gondret F and Quesnel H 2019 Sow environment during gestation: part I. Influence on maternal physiology and lacteal secretions in relation with neonatal survival. *Animal* 13: 1432–1439. 10.1017/S1751731118002987 30468144

[r47] Merlot E, Quesnel H and Prunier A 2013 Prenatal stress, immunity and neonatal health in farm animal species. Animal 7: 2016–2025. 23915487 10.1017/S175173111300147X

[r48] Meyer JS and Novak MA 2012 Mini review: hair cortisol: a novel biomarker of hypothalamic-pituitary-adrenocortical activity. Endocrinology 153: 4120–4127. 22778226 10.1210/en.2012-1226 PMC3423616

[r49] Millet S, Cox E, Buyse J, Goddeeris B and Janssens G 2005 Immunocompetence of fattening pigs fed organic versus conventional diets in organic versus conventional housing. The Veterinary Journal 169: 293–299. 15727924 10.1016/j.tvjl.2004.03.012

[r50] Murata H 2007 Stress and acute phase protein response: an inconspicuous but essential linkage. The Veterinary Journal 3: 473–474. 10.1016/j.tvjl.2006.05.008 16807009

[r51] O’Neill L 2022 *Investigation of antimicrobial use on Irish pig farms and effects on antimicrobial resistance in* Escherichia coli *and the faecal resistome throughout the production cycle*. School of Veterinary Medicine, University College Dublin, Ireland.

[r52] Ortín-Bustillo A, Escribano D, López-Arjona M, Botia M, Fuentes P, Martínez-Miró S, Rubio CP, García-Manzanilla E, Franco-Martínez L and Pardo-Marín L 2022 Changes in a comprehensive profile of saliva analytes in fattening pigs during a complete productive cycle: A longitudinal Study. Animals 12: 1865. 35883410 10.3390/ani12141865 PMC9312009

[r53] Otten W, Heimbürge S, Kanitz E and Tuchscherer A 2020 It’s getting hairy–External contamination may affect the validity of hair cortisol as an indicator of stress in pigs and cattle. General and Comparative Endocrinology 295: 113531. 10.1016/j.ygcen.2020.113531 32535171

[r54] Parada Sarmiento M, Bernardino T, Tatemoto P, Polo G and Zanella AJ 2021 The in-utero experience of piglets born from sows with lameness shapes their life trajectory. Scientific Reports 11: 1–11. 34158529 10.1038/s41598-021-92507-2 PMC8219680

[r55] Quesnel H, Pere MC, Louveau I, Lefaucheur L, Perruchot MH, Prunier A, Pastorelli H, Meunier-Salaun MC, Gardan-Salmon D, Merlot E and Gondret F 2019a Sow environment during gestation: part II. Influence on piglet physiology and tissue maturity at birth. *Animal* 13: 1440–1447. 30442216 10.1017/S1751731118003087

[r56] Quesnel H, Peuteman B, Pere MC, Louveau I, Lefaucheur L, Perruchot MH, Prunier A, Meunier-Salaun MC, Gardan-Salmon D, Gondret F and Merlot E 2019b Effect of environmental enrichment with wood materials and straw pellets on the metabolic status of sows during gestation. Livestock Science 229: 43–48. 10.1016/j.livsci.2019.09.005

[r57] Rault JL, Mack LA, Carter CS, Garner JP, Marchant-Forde JN, Richert BT and Lay DC 2013 Prenatal stress puzzle, the oxytocin piece: prenatal stress alters the behaviour and autonomic regulation in piglets, insights from oxytocin. Applied Animal Behaviour Science 148: 99–107. 10.1016/j.applanim.2013.07.001

[r58] Reimert I, Rodenburg TB, Ursinus WW, Kemp B and Bolhuis JE 2014 Selection based on indirect genetic effects for growth, environmental enrichment and coping style affect the immune status of pigs. PLoS ONE 9: e108700. 10.1371/journal.pone.0108700 PMC4183536 25275507

[r59] Rooney HB, O’Driscoll K, O’Doherty JV and Lawlor PG 2020 Effect of increasing dietary energy density during late gestation and lactation on sow performance, piglet vitality, and lifetime growth of offspring. Journal of Animal Science 98: skz379. 10.1093/jas/skz379 PMC6986436 31875421

[r60] Salaberger T, Millard M, El Makarem S, Mostl E, Grunberger V, Krametter-Frotscher R, Wittek T and Palme R 2016 Influence of external factors on hair cortisol concentrations. General and Comparative Endocrinology 233: 73–78. 10.1016/j.ygcen.2016.05.005 27167500

[r61] Salak-Johnson JL and McGlone JJ 2007 Making sense of apparently conflicting data: stress and immunity in swine and cattle. Journal of Animal Science 85: E81–E88. 10.2527/jas.2006-538 17085721

[r62] Schmitt O, Baxter EM, Lawlor PG, Boyle LA and O’Driscoll K 2019 A single dose of fat-based energy supplement to light birth weight pigs shortly after birth does not increase their survival and growth. Animals 9: 227. 31075904 10.3390/ani9050227 PMC6562461

[r63] Stewart CL, O’Connell NE and Boyle L 2008 Influence of access to straw provided in racks on the welfare of sows in large dynamic groups. Applied Animal Behaviour Science 112: 235–247. 10.1016/j.applanim.2007.09.006

[r64] Tuchscherer M, Kanitz E, Otten W and Tuchscherer A 2002 Effects of prenatal stress on cellular and humoral immune responses in neonatal pigs. Veterinary Immunology and Immunopathology 86: 195–203. 10.1016/S0165-2427(02)00035-1 12007885

[r65] Tuyttens FAM 2005 The importance of straw for pig and cattle welfare: a review. Applied Animal Behaviour Science 92: 261–282.

[r66] van Dixhoorn ID, Reimert I, Middelkoop J, Bolhuis JE, Wisselink HJ, Groot Koerkamp PW, Kemp B, and Stockhofe-Zurwieden N 2016 Enriched housing reduces disease susceptibility to co-infection with porcine reproductive and respiratory virus (PRRSV) and *Actinobacillus pleuropneumoniae* (*A. pleuropneumoniae*) in young pigs. PLoS ONE 11: e0161832. 10.1371/journal.pone.0161832 PMC5015855 27606818

[r67] Wang Y, Kinzie E, Berger FG, Lim S-K and Baumann H 2001 Haptoglobin, an inflammation-inducible plasma protein. Redox Report 6: 379–385. 11865981 10.1179/135100001101536580

[r68] Weinstock M 1997 Does prenatal stress impair coping and regulation of hypothalamic-pituitary-adrenal axis? Neuroscience & Biobehavioral Reviews 21: 1–10. 8994205 10.1016/s0149-7634(96)00014-0

[r69] Winkel C, von Meyer-Höfer M and Heise H 2020 Understanding German pig farmers’ intentions to design and construct pig housing for the improvement of animal welfare. Animals 10: 1760. 32998317 10.3390/ani10101760 PMC7600590

[r70] Xiong Y, Cao S, Xiao H, Wu Q, Yi H, Jiang Z and Wang L 2022 Alterations in intestinal microbiota composition coincide with impaired intestinal morphology and dysfunctional ileal immune response in growing-finishing pigs under constant chronic heat stress. Journal of Animal Science and Biotechnology 13: 1–18. 34983683 10.1186/s40104-021-00651-6 PMC8728975

[r71] Xu J, Li Y, Yang Z, Li C, Liang H, Wu Z and Pu W 2018 Yeast probiotics shape the gut microbiome and improve the health of early-weaned piglets. Frontiers in Microbiology 9: 2011. 30210480 10.3389/fmicb.2018.02011 PMC6119770

